# Comparative Evaluation of Machine Learning Strategies for Analyzing Big Data in Psychiatry

**DOI:** 10.3390/ijms19113387

**Published:** 2018-10-29

**Authors:** Han Cao, Andreas Meyer-Lindenberg, Emanuel Schwarz

**Affiliations:** Department of Psychiatry and Psychotherapy, Central Institute of Mental Health, Medical Faculty Mannheim, Heidelberg University, 68159 Mannheim, Germany; han.cao@zi-mannheim.de (H.C.); Andreas.Meyer-Lindenberg@zi-mannheim.de (A.M.-L.)

**Keywords:** multi-task learning, machine learning, biomarker discovery, psychiatry

## Abstract

The requirement of innovative big data analytics has become a critical success factor for research in biological psychiatry. Integrative analyses across distributed data resources are considered essential for untangling the biological complexity of mental illnesses. However, little is known about algorithm properties for such integrative machine learning. Here, we performed a comparative analysis of eight machine learning algorithms for identification of reproducible biological fingerprints across data sources, using five transcriptome-wide expression datasets of schizophrenia patients and controls as a use case. We found that multi-task learning (MTL) with network structure (MTL_NET) showed superior accuracy compared to other MTL formulations as well as single task learning, and tied performance with support vector machines (SVM). Compared to SVM, MTL_NET showed significant benefits regarding the variability of accuracy estimates, as well as its robustness to cross-dataset and sampling variability. These results support the utility of this algorithm as a flexible tool for integrative machine learning in psychiatry.

## 1. Introduction

Biological research on psychiatric illnesses has highlighted the scale of investigations required to identify reproducible hallmarks of illness [1,2]. In schizophrenia, collaborative analysis of common genetic variants has exceeded 150,000 subjects [3], demonstrating the challenges tied to low-effect sizes of individual variants, large biological and clinical heterogeneity, and genetic complexity. Not surprisingly, these challenges are also found in other mental illnesses [4] and do not seem to be modality specific, as analysis of neuroimaging data, for example, faces similar problems [5,6]. 

The combined “mega-analysis” of data across cohorts and modalities has advantages compared to the more traditional meta-analysis [4,7], as it makes data amenable for a broader spectrum of computational analyses and allows consideration of confounders across studies. There is growing consensus that advanced computational strategies are required to extract biologically meaningful patterns from these data sources. Beyond functional analysis, a particular focus is on machine learning, which, in other areas, has shown substantial success in integrating weak signals into accurate classifiers [8]. In addition to potential clinical use of such classifiers, the discovery of robust biological patterns may uncover new insights into etiological processes. However, the increasing scale and complexity of big data in psychiatry requires careful evaluation of the most suitable computational strategies. A particularly intuitive and very timely problem is the optimal integration of multi-cohort data, where simple concatenation of datasets may give suboptimal results, and even more so when integration is performed across modalities. 

The application of machine-learning techniques on biological problems in psychiatry has already yielded impressive results, including on the prediction of genetic risk, the identification of biomarker candidates, or the exploration of etiological mechanisms [9]. For example, the use of a Bayesian approach for the incorporation of linkage disequilibrium (LD) information during polygenic risk score determination led to a 5% improvement of accuracy in a large schizophrenia dataset [10]. In a study exploring the molecular basis of psychiatric comorbidity, an iterative LASSO approach was used for cross-tissue prediction and identified a schizophrenia expression signature that predicted a peripheral biomarker of T2D [11]. Beyond the analysis of individual data modalities, several machine-learning strategies have been developed for integrative multimodal analysis. For example, a study focusing on the IMAGEN cohort [12] applied an elastic net model to explore information patterns linked to binge drinking across multiple domains, including brain structure and function, personality traits, cognitive differences, candidate gene information, environmental factors, and life experiences. Similarly, another study [13] explored the inherent data sparsity of neuroimaging and psychiatric symptom data, and successfully stratified subjects using sparse canonical correlation analysis. The study found four dimensions of psychopathology with different patterns of connectivity. In the present study, we were particularly interested in multi-task learning (MTL), which aims to improve generalizability by simultaneously learning multiple tasks (such as case-control associations in different datasets) and these learning processes exchange information to achieve a globally optimal solution [14]. Historically, MTL was developed as an extension of neural networks [14], and has since been used across data-intensive research areas, including biomedical informatics [15,16,17,18,19,20], speech and natural language processing [21,22], image processing and computer vision [23,24], and web based applications [25,26]. In psychiatric research, MTL has been applied for integrating measures of cognitive functioning and structural neuroimaging [27], as well as for improved fMRI pattern recognition [28]. In other research fields, MTL approaches have been proposed to combine different sources of biological data, including the linking of MRI or expression with genetic data [29,30], as well as the integrative analysis of multi-cohort expression data [31]. 

In the present study, we used MTL to differentiate schizophrenia patients from controls across multiple transcriptome-wide expression datasets. We hypothesized that MTL is particularly suited for this task, since it allows the consideration of different cohorts as separate classification tasks. As MTL aims to identify predictive patterns that are shared across tasks, it should uncover expression patterns that are biologically reproducible across cohorts. This may result in better and biologically more relevant classifiers compared to those derived from conventional single task learning (STL), which may be unduly influenced by strong signals present in individual cohorts. To test this, we performed a comparative analysis of different MTL and STL approaches in five transcriptome-wide datasets of schizophrenia brain expression. A ‘leave-dataset-out’ procedure was applied to explore and compare the generalizability of the models, with specific focus on classification accuracy, and variability thereof, as well as model sensitivity to cross-dataset and sampling variability.

## 2. Results

### 2.1. Accuracy Comparison Between MTL and STL

**Figure 1:**
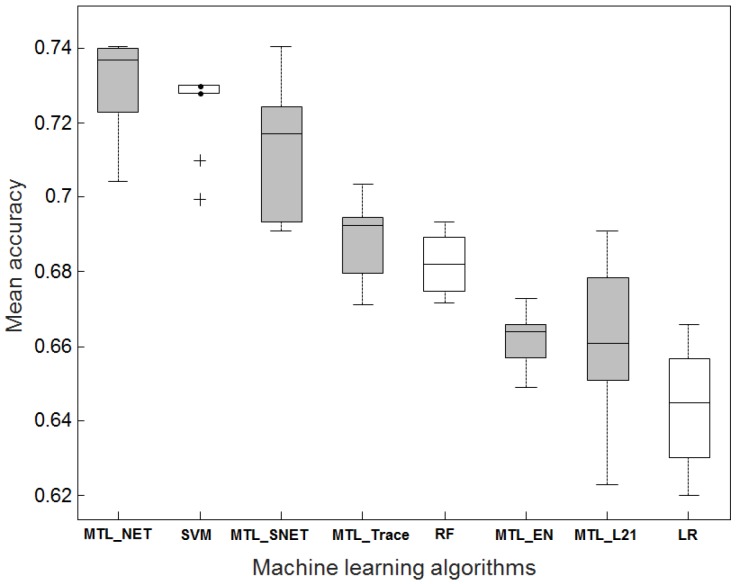
Predictive performance comparison between eight algorithms. The ‘leave-dataset-out’ procedure was used for comparison. Four out of five datasets were combined for training, and then the model was tested on the remaining dataset. The distribution of accuracy estimates indicated the variation of parameter selection across 10 repetitions. The boxplots in gray denote the multi-task learning algorithms.

Figure 1 shows a comparison of average classification accuracies when four out of five datasets were used for training and the remaining dataset for testing. The distributions of accuracies are shown for 10 repetitions of the classification procedure to assess the variability caused by parameter tuning via cross-validation. With an average accuracy of 0.73, MTL_NET outperformed all other methods, followed by SVM, which had a marginally inferior accuracy of 0.72. Moderate accuracies were observed for MTL_Trace (0.69), MTL_L21 (0.66) and RF (0.68). The sparse logistic regression performed worst (0.64). As an extension of MTL_NET and MTL_L21, respectively, MTL_SNET (0.71) and MTL_EN (0.66) achieved similar accuracies to their original algorithms. In the following analysis, we focused on the comparison of MTL_NET and SVM as representatives of MTL and STL, respectively.

In Figure 1, the standard error of accuracies for SVM (0.011) was slightly smaller than that for MTL_NET (0.012), indicating that SVM might be more robust regarding parameter selection. A possible reason was that SVM obtained higher statistical power by comparing cases and controls across datasets. In contrast, MTL_NET derived transcriptomic signatures using cases and controls within datasets, limiting the statistical power.

### 2.2. Dependency of Classification Performance on the Number of Training Datasets 

**Figure 2:**
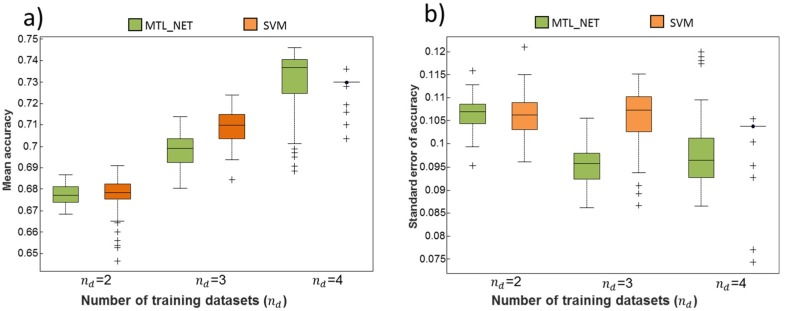
Distribution of classification accuracies and their standard errors across different numbers of training datasets. The Figure shows the mean (**a**) and standard error (**b**) of classification accuracies obtained for different numbers of training datasets ($n_{d}$). Performance was evaluated from the test datasets not used for training. The variation of the boxplot was due to the sampling variability during cross-validation.

We performed a side-by-side comparison of MTL_NET and SVM to explore the dependency of classification performance on the number of available training datasets. Figure 2a shows that increasing accuracy was observed for both MTL_NET and SVM with increasing numbers of training datasets. Notably, MTL_NET only outperformed SVM at *n_d_* = 4 (four datasets used for training), suggesting that MTL required a higher dataset number to identify a reproducible biological pattern. However, we observed that the variation of accuracies for MTL_NET substantially decreased with increasing numbers of training datasets (Figure 2b), which was not the case for SVM. This suggested that MTL_NET was more conservative in that accuracy was not driven by highly successful prediction on an individual test set, but by improved predictability observed for all test sets.

### 2.3. Consistency and Stability of Trained Models

**Figure 3:**
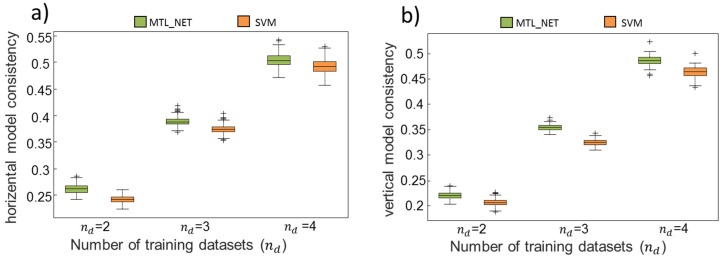
Horizontal and vertical model consistency. To analyze the consistency of a given machine-learning algorithm against the cross-dataset variability, we quantified the horizontal (**a**) and vertical (**b**) model consistency for different numbers (*n_d_*) of training datasets. Specifically, horizontal consistency quantified the similarity between models trained using the same number of datasets, and vertical consistency quantified the pairwise similarity of models, where one was trained using all datasets and the other was trained using less datasets. Stratified 100-fold bootstrapping procedure was applied to quantify the variation of the consistency.

Figure 3a,b show that, in terms of vertical and horizontal consistency, MTL_NET outperformed SVM, independently of the number of training datasets. This indicated that similar discriminative patterns of genes were identified by MTL across training datasets, and implied strong robustness against cross-dataset variability. In particular, the superior performance of vertical consistency for MTL_NET showed that this algorithm was less sensitive to the small numbers of training datasets compared to SVM. Table 1 shows the mean consistency (both horizontal and vertical) across bootstrapping samples. Compared to SVM, MTL_NET achieved a higher mean consistency by approximately 1.6% for horizontal and 2.2% for vertical consistency. Notably, the success rate of consistency was 100%, independent of the number of training sets, showing that MTL_NET models consistently identified higher transcriptomic profile robustness across bootstrapping samples than SVM. 

**Figure 4:**
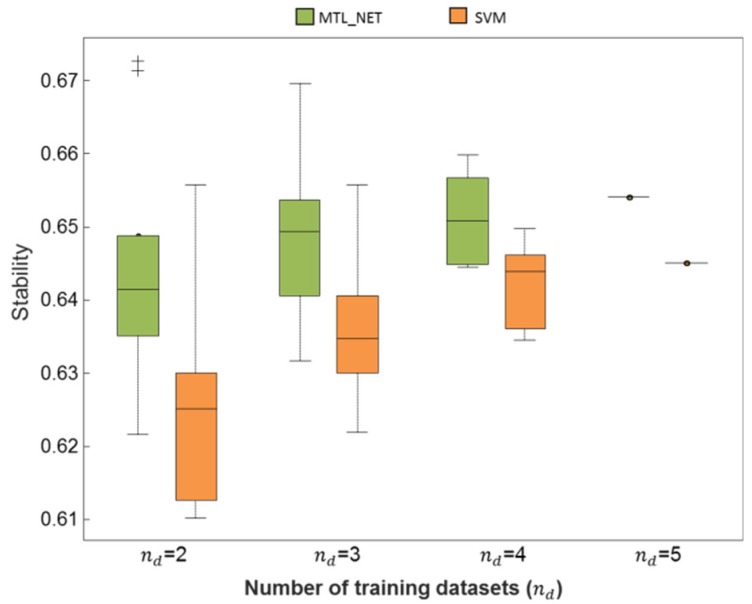
Stability comparison. The stability quantified the robustness of an algorithm against sampling variability. For each $n_{d}$, stability was computed as the pairwise similarity of models trained from two given bootstrap samples. The stability was then averaged across bootstrap samples. In the Figure, the distribution of the stability was due to the different combination of training datasets given, $n_{d}$.

To further identify the robustness of models against sampling variability, we quantified the algorithms’ stability. In Figure 4, across the number of training datasets, *n_d_*, the increasing trend of stability demonstrated that both MTL_NET and SVM gained more robustness against sampling variability with an increasing number of subjects used for training. However, MTL_NET demonstrated higher stability than SVM independently of the number of training datasets (Figure 4). The mean stability across models also supported the result (Table 1). Moreover, the mean stability for MTL_NET was 1.2% higher than SVM (100% success rate of stability across all *n_d_*, Table 2).

We did not perform comparative functional analysis of markers identified by the two algorithms, since marker sets were quite similar. For example, using all five datasets for training, the average similarity over all bootstrapping samples was 98.75%, suggesting that similar functional implications would be derived for these algorithms.

## 3. Discussion

The present study provides a comparative evaluation of using MTL for integrative machine learning, compared to classical, single task learning in five transcriptome-wide datasets of schizophrenia brain expression. Overall, MTL showed similar accuracy, albeit with lower variability, compared to STL. Accuracy estimates varied by up to approximately 10% between algorithms, suggesting different sensitivities of algorithms to cross-dataset heterogeneity as well as sampling variability. Among all MTL formulations, MTL_NET was most predictive. This was likely due to the fact that it harmonized algorithms across tasks with respect to both predictor weight and sign of diagnosis association, resulting in biologically plausible predictive patterns. In contrast, MTL_L21 ignores the sign of association and MTL_Trace improves models’ correlation in each subspace, but failed to modulate the cross-subspace correlation. Contrary to the usual assumption that simpler models show improved generalizability [32], a sparse version of MTL_NET (MTL_SNET) did not improve the prediction. This may be due to the fact that the sparse model was trained by constructing a solution tree among an unlimited number of optimal solution trees. Although these solution trees have similar performance on the training dataset, they may show differently predictive ability on a cross-modality test dataset because the “independent and identically distributed (i.i.d)” assumption may not hold. MTL_NET (as well as SVM) solves a strictly convex optimization problem, resulting in a uniform solution in the entire feature space, which may be equally effective when tested on independent test data.

The higher consistency and stability of MTL_NET implied that a set of similar differentially expressed genes were identified for multiple training datasets. In addition, these genes demonstrated higher predictability and robustness against study-specific effects, which is particularly important for data integration in multi-modal analyses, such as the integrative analysis of genetic and expression data [33] or the analysis of shared markers across multiple comorbid conditions [34,35,36]. 

An interesting observation of the present study was that for MTL_NET, the variance of the classification accuracy substantially decreased with an increasing number of training datasets. This suggested that MTL_NET selected biological signatures with similar effect sizes across independent training datasets, further supporting the biological reproducibility of the identified patterns. In contrast, SVM did not show a decreasing accuracy variance with increasing numbers of training datasets. This indicates that despite the increasing classification accuracy, the identified signatures worked well only for some, but not other, test datasets. These results for these particular datasets highlight differences between single and multi-task learning regarding the variance of the test-set accuracy, which is a fundamentally important consideration for study design and interpretation of classifier reproducibility. 

## 4. Materials and Methods

### 4.1. Datasets

In the present study, five transcriptome-wide expression datasets from schizophrenia post-mortem brains and controls were used for analysis. Details of the datasets are shown in Table 2. All datasets were downloaded from the GEO (Gene Expression Omnibus).

### 4.2. Preprocessing

Preprocessing was performed using the statistical software, R (https://cran.r-project.org/). First, raw expression data were read using the ‘ReadAffy’ function. Then RMA (Multi-Array Average [42]) was applied for background correction, quantile normalization, and log_2_-transformation. Subsequently, multiple probes associated to one gene symbol were averaged. This was followed by the selection of common genes across all datasets (17,061 genes). For each dataset, propensity score matching was used to obtain a sample with approximate 1:1 matching for diagnosis, sex, ph, age, and post-mortem interval (pmi). Next, all datasets were concatenated for quantile normalization and covariate correction. Specifically, the ‘Combat’ function from the R library *sva* [43] was applied to correct for covariates (sex, ph, age, age^2^, pmi, and a dataset indicator). Finally, datasets were separated again for feature standardization (z-score) to remove bias from the expressed genes with large variance and for downstream machine learning analysis.

### 4.3. Machine Learning Approaches

For MTL, multiple across-task regularization strategies were tested, such as MTL with network structure (MTL_NET), sparse network structure (MTL_SNET), joint feature learning (MTL_L21), joint feature learning with elastic net (MTL_EN), and low-rank structure (MTL_Trace). As a comparison, we selected logistic regression with lasso (LR), linear support vector machines (SVM), and random forests (RF) as representatives of conventional STL methods. For all models (except for RF), stratified five-fold cross validation was used to select hyper-parameters. Methodological details of the respective methods are described below. All machine-learning analyses were performed using Matlab (R2016b).

#### 4.3.1. Multi-Task Learning

For all MTL formulations, the logistic loss (L(·)) was used as the common loss function.

(1) $$\;L\left(W,C\right)=\frac{1}{n_{i}}\overset{n_{i}}{\underset{j=1}{\sum }}\log\left(1+e^{\left(-Y_{i,j}\left(X_{i,j}W_{i}^{T}+C_{i}\right)\right)}\right)\;\;$$

where X, Y, W, and C referred to the gene expression matrixes, diagnostic status, weight vectors, and constants of all tasks, respectively. In addition, i and j denoted the index of the dataset and subject respectively, i.e., $n_{i}$ and $W_{i}^{T}$ referred to the number of subject and weight vector of task i. This model aimed to estimate the effect size of each feature such that the likelihood (i.e., the rate of successful prediction in the training data) was maximized. During the prediction procedure, given the expression profile of a previously unseen individual, the model calculates the probability of belonging to the schizophrenia class (with subjects where the probability exceeded 0.5 being assigned to the patient group). Notably, while we focused on classification due to the categorical outcomes of the investigated datasets, the cross-task regularization strategies explored in the present study are not limited to classification, but can also be applied for regression. All MTL formulations were used as implemented in the Matlab library, Malsar [44], or based on custom Matlab implementations. 

(2) $$\;\underset{W,\;C}{\min}\overset{t}{\underset{i=1}{\sum }}L\left(W,C\right)+\lambda \overset{t}{\underset{i=1}{\sum }}{\left|\left|W_{i}-\frac{1}{t}\overset{t}{\underset{j=1}{\sum }}W_{j}\right|\right|}_{2}^{2}\;\;$$

We selected the mean-regularized multi-task learning method [45] as an algorithm for the MTL_NET framework. This algorithm assumes that a latent model exists underlying all tasks, which can be estimated as the mean model across tasks. Based on this assumption, the formulation attempts to identify the most discriminative pattern in the high-dimensional feature space, while limiting the dissimilarity between pairwise models. Dissimilarity is quantified with respect to the effect size of a given predictor and the sign of its association with diagnosis. We expected this combined dissimilarity measure to lead to biologically plausible predictive patterns that are characterized by consistent differences across tasks, both in terms of magnitude as well as directionality. Here, λ had a range of $10^{\left(-6:1:2\right)}$.

(3) $$\;\underset{W,\;C}{\min}\overset{t}{\underset{i=1}{\sum }}L\left(W,C\right)+\lambda \left(\alpha \overset{t}{\underset{i=1}{\sum }}{\left|\left|W_{i}-\frac{1}{t}\overset{t}{\underset{j=1}{\sum }}W_{j}\right|\right|}_{2}^{2}+\left(1-\alpha \right){\left|\left|W\right|\right|}_{1}\right)\;$$

MTL_SNET was the sparse version of MTL_NET, and the sparsity was introduced by the $l_{1}$ norm (i.e., coefficients of predictors with low utility are set to 0). Here, λ controls the entire penalty and α distributes the penalty to full-sparse and non-sparse terms. λ had a range of $10^{\left(-6:1:2\right)}$ and α was chosen from the range [0:0.1:1]. 

(4) $$\;\underset{W,C}{\min}\overset{t}{\underset{i=1}{\sum }}L\left(W,C\right)+\lambda {\left|\left|W\right|\right|}_{2,1}\;$$

The formulation of MTL_L21 introduced the group sparse term, $\;{\left|\left|W\right|\right|}_{2,1}=\overset{p}{\underset{i=1}{\sum }}{\left|\left|W_{i}\right|\right|}_{2}$, which aimed to select or reject the same group of genes across datasets. λ controlled the level of sparsity with a range of $10^{\left(-6:0.1:0\right)}$.

(5) $$\;\underset{W,\;C}{\min}\overset{t}{\underset{i=1}{\sum }}L\left(W,C\right)+\lambda \left(\left(1-\alpha \right){\left|\left|W\right|\right|}_{2,1}+\alpha {\left|\left|W\right|\right|}_{2}^{2}\right)\;$$

The MTL_EN was formulated by adding the composite penalties, where ${\left|\left|W\right|\right|}_{2}^{2}$ is the squared Frobenius norm. Similar to elastic net in conventional STL, such regularization helped to stabilize the solution when multiple highly correlated genes existed in the high-dimensional space [46]. Here, λ had a range of $10^{\left(-6:0.1:0\right)}$ and α was chosen from the range [0:0.1:1].

(6) $$\;\underset{W,\;C}{\min}\overset{t}{\underset{i=1}{\sum }}L\left(W,C\right)+\lambda {\left|\left|W\right|\right|}_{*}\;$$

MTL_Trace encouraged a low-rank model, W, by penalizing the sum of its eigenvalues$,\;\;{\left|\left|W\right|\right|}_{*}$. λ had a range of$\;10^{\left(-6:0.1:1\right)}$. By compressing the subspace spanned by weight vectors, models were structured (i.e., clustered structure). Thus, the models that were clustered together demonstrated high pairwise correlation. 

#### 4.3.2. Conventional, Single-Task Machine Learning

LR_L1: We trained logistic regression with lasso using the package, “Glmnet”. The lambda parameter was chosen among the set, $10^{\left(-10:0.5:1\right)}$.

SVM: Linear support vector machine was trained using the built-in Matlab function, ‘fitcsvm’, with the box constraints in the range of $10^{\left(-5:1:5\right)}$. We only used the linear kernel to facilitate determination of predictor importance.

RF: We used the Matlab built-in function, ‘TreeBagger’, to train a random forest model with 5000 trees. The predictor importance was calculated according to the average error decrement for all splits on a given predictor.

#### 4.3.3. Assessment of Predictive Performance

To quantify predictive performance and capture stability of decision rules against cross-dataset and sampling variability, we used a leave-dataset-out procedure. Specifically, the set of five expression datasets was denoted as $D=\left\{d_{1},d_{2},\ldots ,d_{5}\right\}$ and we calculated the power set, P(D), of D. Then for each subset, d∈P(D), we trained a given algorithm on d and tested the model on D−d. For example, for$\;d=\left\{d_{1},d_{2}\right\}$, we trained using the combination of datasets, $\left\{d_{1},d_{2}\right\}$, and then tested on$\;\{d_{3},d_{4},\;d_{5}\}$. For convenience, we organized these training procedures according to the size of d, noted as $n_{d}\in \left\{2,3,\ldots ,5\right\}$. We thus obtained a series of models trained using all subsets of the five datasets (except for single dataset) and they are referred to using $n_{d}$. 

The comparison of the predictive performance between methods was mainly based on $n_{d}=4$, i.e., when all, but one, datasets were used for training. To understand how dataset-specific confounders affect the prediction, models were trained on a range of $n_{d}$ from 2 to 4. Finally, to explore the convergence of genes’ coefficients across different training datasets, we compared the models trained when $n_{d}=i,\;i\in \left\{2,3\ldots 5\right\}$. 

**Figure A1:**
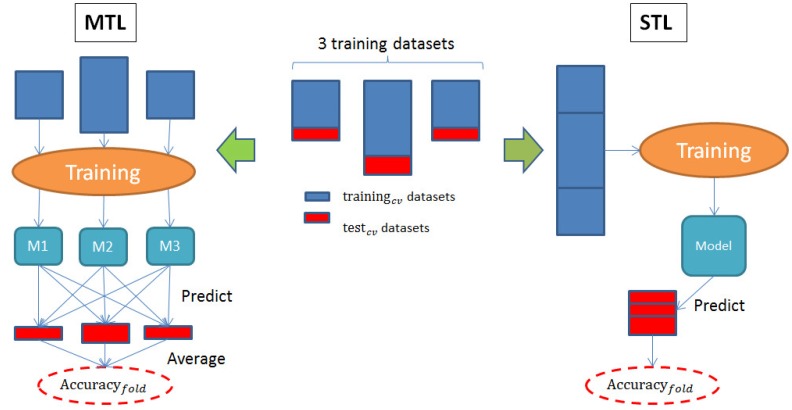
Procedure of five-fold-stratified-cross-validation for Single Task Learning (STL) and Multitask Learning (MTL) (showing one-fold as an example). Using $n_{d}=3\;$as an example, the specific procedure of the cross-validation procedure is shown. First, the subjects were randomly allocated to five folds, stratified for diagnosis per dataset. Subsequently, different strategies were specified for MTL and STL. For MTL, the training datasets were trained in parallel, and the three models (M1, M2, and M3) were tested on each test dataset by averaging the prediction score. The average across all accuracies was used as the final accuracy for the current fold. In contrast, for STL, the training datasets were combined to train a single algorithm that was then predicted on the combined test datasets.

During cross-validation (CV), as illustrated in Figure A1, subjects were randomly allocated to 5 folds, stratified for diagnosis and the dataset indicator. Subsequently, different strategies were specified for MTL and STL. For MTL, the ${\mathrm{training}}_{cv}$ datasets were trained in parallel, and the models were tested on each ${\mathrm{test}}_{cv}$ dataset by averaging the prediction scores. To determine the final accuracy of the current fold, the accuracies retrieved from all ${\mathrm{test}}_{cv}$ datasets were averaged. For STL, the ${\mathrm{training}}_{cv}$ datasets were combined to train a single algorithm that was then predicted on the combined ${\mathrm{test}}_{cv}$ datasets. Similar to CV, in the training procedure, MTL trained on datasets in parallel, while combining the prediction scores for testing. 

#### 4.3.4. Consistency and Stability Analysis

To compare the consistency and stability of markers between algorithms, we used the correlation coefficient as the similarity measure of pairwise transcriptomic profiles (i.e., the coefficient vector for all genes) learnt by algorithms. A high similarity between profiles implied that models shared important predictors with respect to their weights and signs. Using this similarity measure, ‘consistency’ and ‘stability’ were defined, respectively. These measures were derived from 100-fold stratified bootstrapping of subjects from a set of datasets. In each bootstrapping sample, we tested across the number of training sets ($n_{d}=i,\;i\in \left\{2,3,\ldots ,5\right\}$). For MTL, since the training procedure would output multiple coefficient vectors (i.e., training on three datasets would output three coefficient vectors), to compare the similarity between algorithms, the coefficient vectors were averaged. 

**Figure A2:**
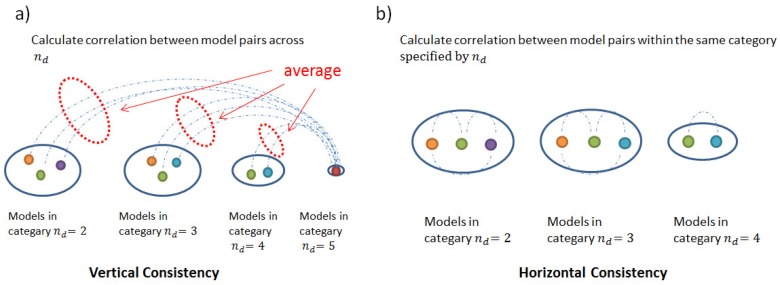
Illustration of model consistency calculation. Consistency quantified the robustness of an algorithm against the cross-dataset variability. To test this, we trained models using each subset of all five expression datasets and then categorized these models according to the number of training sets ($n_{d}$). Different models were rendered as colored circles, categorized by $n_{d}$. For vertical consistency, (**a**) the similarity was determined between the models learned on $n_{d}=2$ to $n_{d}=4$ and the model trained on $n_{d}=5$. The resulting values were then averaged for a given category, $\;n_{d}$. For horizontal consistency, (**b**) the model similarity was calculated in each category, $\;n_{d}$, and then averaged.

**Consistency:** With ‘consistency’, we quantified the pairwise similarity of models trained using overlapping or non-overlapping (i.e., 2 training datasets) datasets. For this, we differentiated two types of consistencies: ‘Horizontal’ and ‘vertical’ consistency as illustrated in Figure A2a,b, respectively. Horizontal consistency quantified model robustness against cross-dataset variability. For this, we fixed the number of training datasets, ($n_{d}$), and determined the pairwise similarity between models. This was performed for all possible choices of $n_{d}$ (see supplementary methods for details). Vertical consistency measured the sensitivity of models to the number of training datasets. For this, we varied $n_{d}$ and quantified similarity between the model determined on all training datasets, ($n_{d}=5)$, and all models derived from lower training datasets numbers, ($n_{d}=i,\;i\in \left\{2,3,4\right\}$) (see supplementary methods for details). Low vertical consistency would, for example, be observed when models trained on two training datasets led to vastly different transcriptomic profiles compared to that using all five datasets for training. 

**Figure A3:**
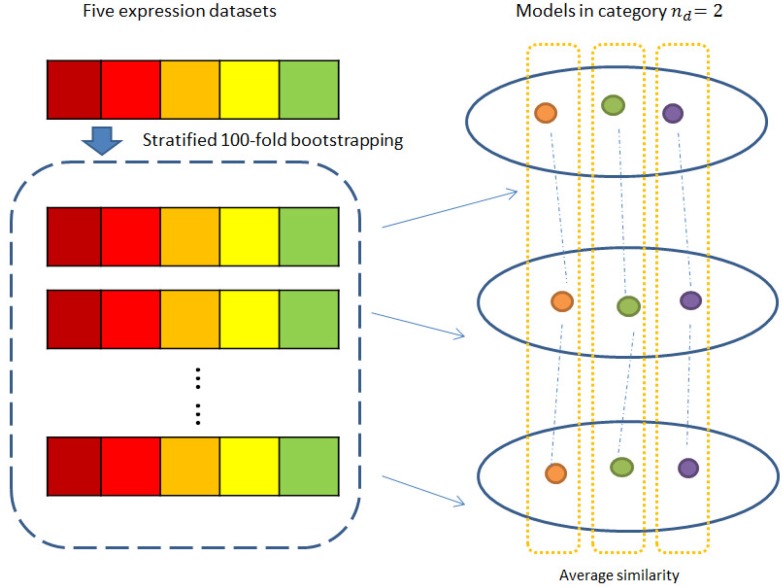
Illustration of model stability calculation. Stability quantified the robustness of an algorithm against sampling variability. This metric was computed by performing 100-fold-stratified-bootstrapping. In the left panel, five expression datasets are shown as colored boxes. Using $n_{d}=2$ as an example, two out of five datasets were combined for training in each bootstrapping sample. Thus, a series of models were obtained as illustrated as the colored circles in the right panel. The stability was determined as the average pairwise similarity for each model, calculated across all pairs of bootstrapping samples.

**Stability:** To quantify the stability of an algorithm against the sampling variability, we observed the variation of transcriptomic profiles learnt from different bootstrapping samples as illustrated in Figure A3. Then the variation of all models given $\;n_{d}$ was summarized as the stability (see supplementary methods for details).

**Success rate:** In addition to consistency and stability, to perform a side-by-side comparison of algorithms, we defined the success rate as the proportion of cases where one algorithm outperformed the other. For example, we quantified the success rate of consistency as the proportion of bootstrapping samples where the first algorithm demonstrated higher consistency than the second (see supplementary methods for details). The success rate of stability was quantified as the proportion of models, which were more stable for the first algorithm than that for the second (see supplementary methods for details). 

## 5. Limitations and Future Work

This work evaluates the performance of MTL and STL for biomarker analysis across five transcriptomic schizophrenia expression datasets. Several quality control procedures were employed to remove unwanted variation in the investigated datasets and to improve the biological generalizability of the obtained results. Despite this, the presented results should be interpreted in the light of the specific datasets investigated. Since other data modalities, including neuroimaging or gene methylation, show similar cross-dataset heterogeneity and correlation structures across variables, the present results may not be limited to expression data, although this remains to be empirically demonstrated. Furthermore, future investigations should include systematic simulation studies to explore the performance of MTL and its robustness against factors typically affecting machine learning performance, including data dimensionality, predictor effect sizes, and biological as well as experimental variability across datasets.

## 6. Conclusions

The present study demonstrates the utility of MTL for integrative machine learning in high-dimensional datasets, compared to classical single-task learning. Mega-analyses that require integration of data across numerous datasets are becoming more frequent, but thus far, have rarely used machine learning approaches. The present study shows that MTL bears substantial promise for such applications. This particularly applies for scenarios where inter-dataset heterogeneity far outweighs the illness associated signal, a typical case for high-dimensional datasets in psychiatric research.

## Figures and Tables

**Table 1:** Mean consistency, stability, and success rate across the number of training sets, $n_{d}$.

MTL_NET/SVM	*n_d_* = 2	*n_d_* = 3	*n_d_* = 4	*n_d_* = 5
Horizontal consistency	**0.26**/0.24	**0.39**/0.37	**0.51**/0.49	-
Vertical consistency	**0.22**/0.21	**0.35**/0.33	**0.49**/0.46	-
Stability	**0.64**/0.63	**0.65**/0.64	**0.65**/0.64	**0.654**/0.645
Success rate (horizontal consistency)	1	1	1	-
Success rate (vertical consistency)	1	1	1	-
Success rate (stability)	1	1	1	1

**Table 2:** Overview of demographic details. Values are shown as mean ± sd.

	GSE12679	GSE35977	GSE17612	GSE21935	GSE21138
Reference	[37]	[38]	[39]	[40]	[41]
n SZ	11	50	22	19	29
n HC	11	50	22	19	29
age SZ	46.1 ± 5.9	42.4 ± 9.9	76 ± 12.9	77.6 ± 11.4	43.3 ± 17.3
age HC	41.7 ± 7.9	45.5 ± 9	68 ± 21.5	67.7 ± 22.2	44.7 ± 16.1
sex SZ (m/f)	7/4	37/13	16/6	11/8	23/6
sex HC (m/f)	8/3	35/15	11/11	10/9	24/5
PMI SZ	33 ± 6.7	31.8 ± 15.4	6.2 ± 4.1	5.5 ± 2.6	38.1 ± 10.8
PMI HC	24.2 ± 15.7	27.3 ± 11.8	10.1 ± 4.3	9.1 ± 4.3	40.5 ± 14
brain pH SZ	NA	6.4 ± 0.3	6.1 ± 0.2	6.1 ± 0.2	6.2 ± 0.2
brain pH HC	NA	6.5 ± 0.3	6.5 ± 0.3	6.5 ± 0.3	6.3 ± 0.2
Genechip	HGU	HuG	HGU	HGU	HGU
Brain Region	PFC	PC	APC	STC	PFC

HGU: HG-U133_Plus_2; HuG = HuGene-1_0-st; APC: Anterior prefrontal cortex; PFC: Prefrontal cortex; PC: Parietal cortex; STC: Superior temporal cortex; HC: Healthy control; SZ: Schizophrenia.

## Abbreviations

MTL	Multi-task learning
STL	Single-task learning
RF	Random Forests
SVM	Support Vector Machine

## Appendix A

**Supplementary Methods**

**Consistency, stability and success rate**

**Notations:**

- The model pairs trained using different (overlapping or non-overlapping) combinations of datasets were represented as M and $\tilde{M}$, respectively (i.e., M represented the model trained using the training set, d={1, 2}; $\tilde{M}$ was trained using a different dataset combination, for example, d={3,4} or d={1, 2,…,5})
- The notation of an algorithm: α, β (i.e., α = MTL_NET, β = SVM)
- The index of the bootstrapping sample: b∈{1,2,…100} and $\tilde{b}\in \left\{1,2,\ldots 100\right\}$. For computational efficiency, bootstrapping was performed across all datasets, d={1, 2,…,5}, and data subsets were selected from this sampling.

As an example, a model, $M_{b}^{\alpha }$, could be trained based on bootstrap sample, b=3, from which training sets, d={1, 2}, were extracted, using the algorithm, α=SVM. The model trained on the same bootstrap sample based on a different combination of training sets and using the algorithm, α=SVM, would be denoted as$\;{\tilde{M}}_{b}^{\alpha }$.

**Consistency**

Given$\;n_{d}=i,\;i\in \left\{2,3,4\right\}$ and the algorithm, α, we calculated the expected similarity for each bootstrapping sample, b as:

$$\;C_{b}^{\alpha ,\;n_{d}}=E_{M,\;\tilde{M},\;M\neq \tilde{M}}⟦Cor(M_{b}^{\alpha },\;{\tilde{M}}_{b}^{\alpha })⟧\;$$

Then, the expected similarity list, $C^{\alpha ,\;n_{d}}=\left[C_{1}^{\alpha ,\;n_{d}},\;C_{2}^{\alpha ,\;n_{d}},\ldots ,C_{100}^{\alpha ,\;n_{d}}\right]$, over b was the consistency list of algorithm, α, for a given $n_{d}$. Here, the expectation was calculated empirically by enumerating all pairs of models, M and $\tilde{M}$. By assigning different values to M and $\tilde{M}$, horizontal and vertical consistency were differentiated. For horizontal consistency, M and $\tilde{M}$ represented the pairwise models trained using the same number ($n_{d}$) of datasets. For vertical consistency, $\tilde{M}$ was trained using $n_{d}=5$ datasets and M was trained using fewer datasets.

**Stability**

Given $n_{d}=i,\;i\in \left\{2,3,4\right\}$, and algorithm, α, we quantified the expected similarity between pairwise models ($M_{b}^{\alpha }$ and $M_{\tilde{b}}^{\alpha }$), which were trained using the same datasets (M), but different bootstrapping samples (b and $\tilde{b}$), as:

$$\;S_{M}^{\alpha ,\;n_{d}}=E_{b,\;\tilde{b},\;b\neq \tilde{b}}⟦Cor\left(M_{b}^{\alpha },\;M_{\tilde{b}}^{\alpha }\right)⟧\;$$

Over all models, (M), $S^{\alpha ,\;n_{d}}=\left[S_{1}^{\alpha ,\;n_{d}},\;S_{2}^{\alpha ,\;n_{d}},\ldots ,S_{\left(\begin{array}{c} 5\\ n_{d} \end{array}\right)}^{\alpha ,\;n_{d}}\right]$ was quantified as the stability list of algorithm, α, given$\;n_{d}$. The expectation was estimated empirically by enumerating all pairs of bootstrapping samples, b and$\;\tilde{b}$.

**Success rate**

The success rate compared algorithms, α and β, side-by-side, and was measured as the proportion of cases where algorithm, α, outperformed β.

For example, given the consistency list of algorithm, α and β, ($C^{\alpha ,\;n_{d}}\;and\;C^{\beta ,\;n_{d}}$), we determined the proportion of bootstrapping samples where algorithm, α, demonstrated higher consistency than β, yielding the success rate of consistency:

$$\;SR_{C}^{n_{d}}=E_{b}⟦1_{C_{b}^{\alpha ,\;n_{d}}-C_{b}^{\beta ,\;n_{d}}>0}⟧\;$$

Given the stability list of algorithm, α and β, ($S^{\alpha ,\;n_{d}}\;and\;S^{\beta ,\;n_{d}}$), we determined the proportion of models, which demonstrated higher stability for algorithm, α, yielding the success rate of stability:

$$\;SR_{S}^{n_{d}}=E_{M}⟦1_{S_{M}^{\alpha ,\;n_{d}}-S_{M}^{\beta ,\;n_{d}}>0}⟧\;$$
